# 3-Bromo-*N*′-[(*E*)-4-hydroxy­benzyl­idene]benzohydrazide

**DOI:** 10.1107/S1600536808015912

**Published:** 2008-06-07

**Authors:** Tao Yang, Guo-Biao Cao, Ji-Ming Xiang, Li-Hui Zhang

**Affiliations:** a Department of Chemistry, Ankang University, Ankang, Shanxi 725000, People’s Republic of China

## Abstract

The title compound, C_14_H_11_BrN_2_O_2_, was synthesized by the reaction of 4-hydroxy­benzaldehyde with an equimolar quantity of 3-bromo­benzohydrazide in methanol. The dihedral angle between the two benzene rings is 40.1 (2)°. In the crystal structure, mol­ecules are linked through inter­molecular O—H⋯O, O—H⋯N and N—H⋯O hydrogen bonds to form a three-dimensional network.

## Related literature

For related structures, see: Cao (2007*a* ▶,*b* ▶).
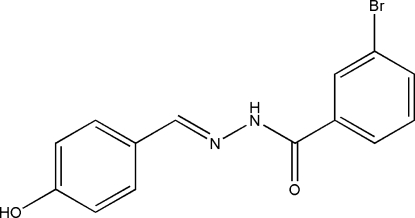


## Experimental

### 

#### Crystal data

C_14_H_11_BrN_2_O_2_
*M* *_r_* = 319.16
Orthorhombic, 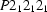

*a* = 7.5576 (11) Å
*b* = 11.7337 (18) Å
*c* = 15.021 (2) Å
*V* = 1332.0 (3) Å^3^
*Z* = 4
Mo *K*α radiation
μ = 3.08 mm^−1^
*T* = 298 (2) K
0.20 × 0.17 × 0.16 mm

#### Data collection

Bruker SMART CCD area-detector diffractometer
Absorption correction: multi-scan (*SADABS*; Bruker, 2001 ▶) *T*_min_ = 0.577, *T*_max_ = 0.638
7740 measured reflections
2757 independent reflections
2145 reflections with *I* > 2σ(*I*)
*R*_int_ = 0.038

#### Refinement

*R*[*F* ^2^ > 2σ(*F* ^2^)] = 0.030
*wR*(*F* ^2^) = 0.059
*S* = 0.97
2757 reflections
176 parameters
1 restraint
H atoms treated by a mixture of independent and constrained refinement
Δρ_max_ = 0.17 e Å^−3^
Δρ_min_ = −0.29 e Å^−3^
Absolute structure: Flack (1983 ▶), with 1154 Friedel pairs
Flack parameter: 0.006 (9)

### 

Data collection: *SMART* (Bruker, 2007 ▶); cell refinement: *SAINT* (Bruker, 2007 ▶); data reduction: *SAINT*; program(s) used to solve structure: *SHELXTL* (Sheldrick, 2008 ▶); program(s) used to refine structure: *SHELXTL*; molecular graphics: *SHELXTL*; software used to prepare material for publication: *SHELXTL*.

## Supplementary Material

Crystal structure: contains datablocks global, I. DOI: 10.1107/S1600536808015912/sj2509sup1.cif

Structure factors: contains datablocks I. DOI: 10.1107/S1600536808015912/sj2509Isup2.hkl

Additional supplementary materials:  crystallographic information; 3D view; checkCIF report

## Figures and Tables

**Table 1:** Hydrogen-bond geometry (Å, °)

*D*—H⋯*A*	*D*—H	H⋯*A*	*D*⋯*A*	*D*—H⋯*A*
O1—H1⋯O2^i^	0.82	1.95	2.750 (2)	166
O1—H1⋯N1^i^	0.82	2.56	3.003 (3)	116
N2—H2*A*⋯O1^ii^	0.904 (10)	2.136 (14)	3.007 (3)	162 (3)

Symmetry codes: (i) 
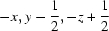
; (ii) 
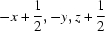
.

**Fig. 1.**
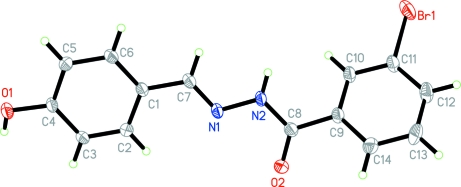
The molecular structure of (I) with ellipsoids drawn at the 30% probability level.

**Fig. 2.**
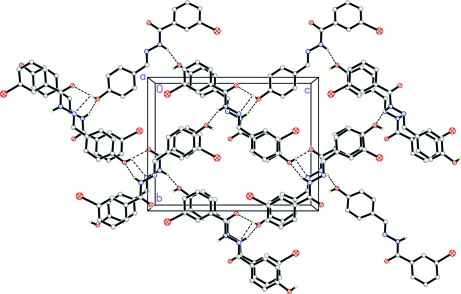
The molecular packing of (I), viewed along the a axis. Hydrogen bonds are drawn as dashed lines.

## supplementary crystallographic information

### Comment

We have recently reported some transition metal complexes with Schiff base ligands (Cao, 2007*a*,*b*). We report herein the crystal structure of the title compound, (I), derived from the reaction of 4-hydroxybenzaldehyde with an equimolar quantity of 3-bromobenzohydrazide in methanol.

In compound (I), Fig. 1, the dihedral angle between the two benzene rings is 40.1 (2)°. In the crystal structure, molecules are linked through intermolecular O—H···O, O—H···N, and N—H···O hydrogen bonds, Table 1, to form a three-dimensional network, Figure 2.

### Experimental

The compound was prepared by refluxing equimolar quantities of 4-hydroxybenzaldehyde with 3-bromobenzohydrazide in methanol. Colourless block-like crystals were formed when the solution was evaporated in air for about a week.

### Refinement

H2A was located in a difference Fourier map and refined isotropically, with the N—H distance restrained to 0.90 (1) Å. The other H atoms were placed in idealized positions and constrained to ride on their parent atoms, with C—H distances of 0.93 Å, the O—H distance 0.82 Å, and with *U*_iso_(H) set at 1.2*U*_eq_(C) and 1.5*U*_eq_(O).

### Crystal data

**Table:**

C_14_H_11_BrN_2_O_2_	*F*_000_ = 640
*M**_r_* = 319.16	*D*_x_ = 1.591 Mg m^−^^3^
Orthorhombic, *P*2_1_2_1_2_1_	Mo *K*α radiation λ = 0.71073 Å
Hall symbol: P 2ac 2ab	Cell parameters from 1827 reflections
*a* = 7.5576 (11) Å	θ = 2.3–24.5º
*b* = 11.7337 (18) Å	µ = 3.09 mm^−^^1^
*c* = 15.021 (2) Å	*T* = 298 (2) K
*V* = 1332.0 (3) Å^3^	Block, colourless
*Z* = 4	0.20 × 0.17 × 0.16 mm

### Data collection

**Table:**

Bruker SMART CCD area-detector diffractometer	2757 independent reflections
Radiation source: fine-focus sealed tube	2145 reflections with *I* > 2σ(*I*)
Monochromator: graphite	*R*_int_ = 0.038
*T* = 298(2) K	θ_max_ = 26.6º
ω scans	θ_min_ = 2.2º
Absorption correction: multi-scan(SADABS; Bruker, 2001)	*h* = −9→9
*T*_min_ = 0.577, *T*_max_ = 0.638	*k* = −10→14
7740 measured reflections	*l* = −18→18

### Refinement

**Table:**

Refinement on *F*^2^	Hydrogen site location: inferred from neighbouring sites
Least-squares matrix: full	H atoms treated by a mixture of independent and constrained refinement
*R*[*F*^2^ > 2σ(*F*^2^)] = 0.031	*w* = 1/[σ^2^(*F*_o_^2^) + (0.0151*P*)^2^] where *P* = (*F*_o_^2^ + 2*F*_c_^2^)/3
*wR*(*F*^2^) = 0.059	(Δ/σ)_max_ = 0.001
*S* = 0.97	Δρ_max_ = 0.17 e Å^−^^3^
2757 reflections	Δρ_min_ = −0.29 e Å^−^^3^
176 parameters	Extinction correction: none
1 restraint	Absolute structure: Flack (1983), with 1154 Friedel pairs
Primary atom site location: structure-invariant direct methods	Flack parameter: 0.006 (9)
Secondary atom site location: difference Fourier map	

### Special details

**Table:**

Geometry. All e.s.d.'s (except the e.s.d. in the dihedral angle between two l.s. planes) are estimated using the full covariance matrix. The cell e.s.d.'s are taken into account individually in the estimation of e.s.d.'s in distances, angles and torsion angles; correlations between e.s.d.'s in cell parameters are only used when they are defined by crystal symmetry. An approximate (isotropic) treatment of cell e.s.d.'s is used for estimating e.s.d.'s involving l.s. planes.
Refinement. Refinement of *F*^2^ against ALL reflections. The weighted *R*-factor *wR* and goodness of fit *S* are based on *F*^2^, conventional *R*-factors *R* are based on *F*, with *F* set to zero for negative *F*^2^. The threshold expression of *F*^2^ > σ(*F*^2^) is used only for calculating *R*-factors(gt) *etc*. and is not relevant to the choice of reflections for refinement. *R*-factors based on *F*^2^ are statistically about twice as large as those based on *F*, and *R*- factors based on ALL data will be even larger.

### Fractional atomic coordinates and isotropic or equivalent isotropic displacement parameters (Å^2^)

**Table:**

	*x*	*y*	*z*	*U*_iso_*/*U*_eq_	
Br1	0.04594 (5)	0.39161 (3)	0.901456 (17)	0.06805 (13)	
O1	0.1347 (3)	−0.14174 (14)	0.15827 (10)	0.0414 (5)	
H1	0.0663	−0.1163	0.1208	0.062*	
O2	0.0446 (3)	0.45672 (13)	0.48129 (10)	0.0435 (4)	
N1	0.1134 (3)	0.23480 (18)	0.46451 (12)	0.0380 (6)	
N2	0.1329 (3)	0.29110 (18)	0.54514 (12)	0.0372 (5)	
C1	0.1401 (3)	0.0602 (2)	0.38450 (14)	0.0310 (6)	
C2	0.0757 (3)	0.1044 (2)	0.30437 (14)	0.0343 (6)	
H2	0.0339	0.1789	0.3024	0.041*	
C3	0.0735 (3)	0.03889 (19)	0.22853 (14)	0.0331 (6)	
H3	0.0315	0.0692	0.1754	0.040*	
C4	0.1341 (3)	−0.0726 (2)	0.23148 (14)	0.0309 (6)	
C5	0.1975 (3)	−0.1177 (2)	0.31015 (15)	0.0357 (6)	
H5	0.2381	−0.1925	0.3120	0.043*	
C6	0.2003 (3)	−0.0514 (2)	0.38585 (15)	0.0352 (6)	
H6	0.2432	−0.0820	0.4387	0.042*	
C7	0.1494 (3)	0.1299 (2)	0.46504 (15)	0.0348 (6)	
H7	0.1829	0.0958	0.5183	0.042*	
C8	0.0976 (3)	0.4039 (2)	0.54681 (14)	0.0339 (6)	
C9	0.1227 (3)	0.4615 (2)	0.63473 (15)	0.0333 (6)	
C10	0.0837 (3)	0.4069 (2)	0.71421 (14)	0.0373 (6)	
H10	0.0457	0.3315	0.7143	0.045*	
C11	0.1020 (4)	0.4657 (2)	0.79290 (15)	0.0405 (7)	
C12	0.1571 (4)	0.5781 (2)	0.79396 (18)	0.0482 (8)	
H12	0.1689	0.6169	0.8476	0.058*	
C13	0.1942 (4)	0.6314 (2)	0.7149 (2)	0.0516 (8)	
H13	0.2320	0.7068	0.7151	0.062*	
C14	0.1760 (4)	0.5743 (2)	0.63504 (17)	0.0425 (7)	
H14	0.1995	0.6116	0.5817	0.051*	
H2A	0.195 (4)	0.257 (2)	0.5890 (15)	0.080*	

### Atomic displacement parameters (Å^2^)

**Table:**

	*U*^11^	*U*^22^	*U*^33^	*U*^12^	*U*^13^	*U*^23^
Br1	0.0960 (3)	0.0793 (2)	0.02888 (14)	0.0102 (2)	0.00631 (17)	−0.00726 (16)
O1	0.0589 (13)	0.0376 (10)	0.0276 (8)	0.0034 (9)	−0.0034 (9)	−0.0093 (8)
O2	0.0655 (12)	0.0350 (10)	0.0301 (8)	0.0023 (10)	−0.0072 (10)	0.0018 (8)
N1	0.0566 (16)	0.0338 (13)	0.0237 (10)	0.0039 (11)	−0.0060 (10)	−0.0063 (9)
N2	0.0581 (16)	0.0306 (13)	0.0230 (11)	0.0104 (11)	−0.0077 (10)	−0.0070 (9)
C1	0.0354 (14)	0.0340 (13)	0.0236 (13)	−0.0019 (11)	−0.0012 (10)	−0.0033 (10)
C2	0.0435 (16)	0.0295 (12)	0.0298 (11)	−0.0011 (13)	0.0013 (11)	−0.0003 (11)
C3	0.0424 (16)	0.0334 (14)	0.0235 (11)	0.0021 (12)	−0.0035 (11)	0.0015 (10)
C4	0.0343 (14)	0.0348 (15)	0.0238 (12)	−0.0046 (11)	0.0011 (11)	−0.0070 (10)
C5	0.0470 (17)	0.0258 (14)	0.0343 (12)	0.0036 (13)	−0.0010 (11)	−0.0011 (12)
C6	0.0452 (16)	0.0362 (14)	0.0243 (13)	0.0032 (11)	−0.0068 (11)	0.0013 (11)
C7	0.0414 (15)	0.0393 (17)	0.0238 (11)	0.0003 (13)	−0.0026 (11)	−0.0027 (11)
C8	0.0370 (16)	0.0361 (15)	0.0287 (12)	−0.0032 (13)	0.0000 (10)	−0.0013 (12)
C9	0.0362 (15)	0.0342 (15)	0.0297 (12)	0.0039 (12)	−0.0045 (11)	−0.0069 (11)
C10	0.0434 (17)	0.0381 (14)	0.0304 (12)	0.0049 (13)	−0.0038 (11)	−0.0097 (12)
C11	0.0450 (18)	0.0456 (17)	0.0308 (13)	0.0094 (13)	−0.0012 (11)	−0.0093 (12)
C12	0.0512 (19)	0.055 (2)	0.0388 (15)	0.0096 (15)	−0.0131 (14)	−0.0196 (14)
C13	0.054 (2)	0.0368 (17)	0.0644 (19)	0.0002 (14)	−0.0103 (15)	−0.0221 (15)
C14	0.0483 (18)	0.0370 (17)	0.0421 (15)	−0.0020 (13)	−0.0031 (13)	−0.0037 (12)

### Geometric parameters (Å, °)

**Table:**

Br1—C11	1.896 (3)	C5—C6	1.378 (3)
O1—C4	1.367 (3)	C5—H5	0.9300
O1—H1	0.8200	C6—H6	0.9300
O2—C8	1.230 (3)	C7—H7	0.9300
N1—C7	1.260 (3)	C8—C9	1.495 (3)
N1—N2	1.387 (2)	C9—C14	1.384 (4)
N2—C8	1.351 (3)	C9—C10	1.387 (3)
N2—H2A	0.904 (10)	C10—C11	1.376 (3)
C1—C6	1.386 (3)	C10—H10	0.9300
C1—C2	1.398 (3)	C11—C12	1.382 (4)
C1—C7	1.462 (3)	C12—C13	1.371 (4)
C2—C3	1.374 (3)	C12—H12	0.9300
C2—H2	0.9300	C13—C14	1.381 (4)
C3—C4	1.386 (3)	C13—H13	0.9300
C3—H3	0.9300	C14—H14	0.9300
C4—C5	1.381 (3)		
			
C4—O1—H1	109.5	N1—C7—H7	119.0
C7—N1—N2	115.88 (19)	C1—C7—H7	119.0
C8—N2—N1	117.51 (19)	O2—C8—N2	122.9 (2)
C8—N2—H2A	121.7 (19)	O2—C8—C9	121.4 (2)
N1—N2—H2A	119 (2)	N2—C8—C9	115.7 (2)
C6—C1—C2	118.5 (2)	C14—C9—C10	120.1 (2)
C6—C1—C7	120.0 (2)	C14—C9—C8	118.2 (2)
C2—C1—C7	121.4 (2)	C10—C9—C8	121.7 (2)
C3—C2—C1	120.7 (2)	C11—C10—C9	119.1 (2)
C3—C2—H2	119.7	C11—C10—H10	120.5
C1—C2—H2	119.7	C9—C10—H10	120.5
C2—C3—C4	119.8 (2)	C10—C11—C12	121.3 (2)
C2—C3—H3	120.1	C10—C11—Br1	119.1 (2)
C4—C3—H3	120.1	C12—C11—Br1	119.65 (19)
O1—C4—C5	117.4 (2)	C13—C12—C11	119.1 (2)
O1—C4—C3	122.4 (2)	C13—C12—H12	120.4
C5—C4—C3	120.2 (2)	C11—C12—H12	120.4
C6—C5—C4	119.7 (2)	C12—C13—C14	120.7 (3)
C6—C5—H5	120.2	C12—C13—H13	119.6
C4—C5—H5	120.2	C14—C13—H13	119.6
C5—C6—C1	121.1 (2)	C13—C14—C9	119.7 (3)
C5—C6—H6	119.5	C13—C14—H14	120.1
C1—C6—H6	119.5	C9—C14—H14	120.1
N1—C7—C1	122.1 (2)		

### Hydrogen-bond geometry (Å, °)

**Table:**

*D*—H···*A*	*D*—H	H···*A*	*D*···*A*	*D*—H···*A*
O1—H1···O2^i^	0.82	1.95	2.750 (2)	166
O1—H1···N1^i^	0.82	2.56	3.003 (3)	116
N2—H2A···O1^ii^	0.904 (10)	2.136 (14)	3.007 (3)	162 (3)

Symmetry codes: (i) −*x*, *y*−1/2, −*z*+1/2; (ii) −*x*+1/2, −*y*, *z*+1/2.
